# *Amblyomma sculptum* Salivary Protease Inhibitors as Potential Anti-Tick Vaccines

**DOI:** 10.3389/fimmu.2020.611104

**Published:** 2021-02-04

**Authors:** Gabriel Cerqueira Alves Costa, Izabela Cosso Tavares Ribeiro, Otoni Melo-Junior, Nelder F. Gontijo, Mauricio R.V. Sant’Anna, Marcos H. Pereira, Grasielle C.D. Pessoa, Leonardo B. Koerich, Fabiano Oliveira, Jesus G. Valenzuela, Rodolfo Cordeiro Giunchetti, Ricardo Toshio Fujiwara, Daniella Castanheira Bartholomeu, Ricardo N. Araujo

**Affiliations:** ^1^ Laboratory of Physiology of Hematophagous Insects, Department of Parasitology, Universidade Federal de Minas Gerais, Belo Horizonte, Brazil; ^2^ Laboratory of Cell-Cell Interactions, Department of Morphology, Universidade Federal de Minas Gerais, Belo Horizonte, Brazil; ^3^ Instituto Nacional de Ciência e Tecnologia em Entomologia Molecular, Rio de Janeiro, Brazil; ^4^ Section of Vector Biology, Laboratory of Malaria and Vector Research, National Institute of Allergy and Infectious Diseases, National Institutes of Health, Bethesda, MD, United States; ^5^ Laboratory of Immunology and Genomics of Parasites, Department of Parasitology, Universidade Federal de Minas Gerais, Belo Horizonte, Brazil

**Keywords:** *Amblyomma sculptum*, saliva, antihemostatic, complement inhibitors, vaccine

## Abstract

*Amblyomma sculptum* is the main tick associated with human bites in Brazil and the main vector of *Rickettsia rickettsii*, the causative agent of the most severe form of Brazilian spotted fever. Molecules produced in the salivary glands are directly related to feeding success and vector competence. In the present study, we identified sequences of *A. sculptum* salivary proteins that may be involved in hematophagy and selected three proteins that underwent functional characterization and evaluation as vaccine antigens. Among the three proteins selected, one contained a Kunitz_bovine pancreatic trypsin inhibitor domain (named AsKunitz) and the other two belonged to the 8.9 kDa and basic tail families of tick salivary proteins (named As8.9kDa and AsBasicTail). Expression of the messenger RNA (mRNA) encoding all three proteins was detected in the larvae, nymphs, and females at basal levels in unfed ticks and the expression levels increased after the start of feeding. Recombinant proteins rAs8.9kDa and rAsBasicTail inhibited the enzymatic activity of factor Xa, thrombin, and trypsin, whereas rAsKunitz inhibited only thrombin activity. All three recombinant proteins inhibited the hemolysis of both the classical and alternative pathways; this is the first description of tick members of the Kunitz and 8.9kDa families being inhibitors of the classical complement pathway. Mice immunization with recombinant proteins caused efficacies against A. *sculptum* females from 59.4% with rAsBasicTail immunization to more than 85% by immunization with rAsKunitz and rAs8.9kDa. The mortality of nymphs fed on immunized mice reached 70–100%. Therefore, all three proteins are potential antigens with the possibility of becoming a new tool in the control of *A. sculptum*.

## Introduction

*Amblyomma sculptum* is the major tick species of medical importance in Brazil. It displays frequent parasitism of humans, high distribution in green areas of populated regions in both rural and urban areas, and is the main vector of *Rickettsia rickettsii*, the bacterium that causes the most frequent and severe form of Brazilian spotted fever (1–4). In addition, *A. sculptum* causes several problems in livestock rearing (5).

The reduction of host and environmental infestations of *A. sculptum* is of the utmost importance to prevent tick bites and pathogen transmission. The main control methods applied are based on the use of acaricides that, despite good results when used correctly, have raised concerns about the risk of contamination of products of animal origin and the environment, as well as the possibility of activity loss from the selection of resistant populations (6). *Amblyomma sculptum* is a three host tick, with a seasonal life cycle and short periods of contact with hosts that vary from 5 to 10 days (7, 8). Therefore, the use of acaricides has to be undertaken more frequently as strategic treatments recommend up to 16 to 28 applications throughout the year at weekly intervals (9). Such a strategy was developed to control ticks on horses, which are the main domestic hosts of *A. sculptum*. However, this treatment becomes extremely costly and even impractical when it needs to be administered on wild animals with serious difficulties regarding capturing, restraining, and application. Much of this concern is directed at the capybaras, which are considered to be the main wild hosts of *A. sculptum*, and can maintain high environmental tick infestations in urban and rural areas and are directly involved in the epidemiology of Brazilian spotted fever (1–3).

Therefore, the development of new tools for the control of *A. sculptum*, which can be applied alone or combined with acaricides, is extremely important. Anti-tick vaccines have emerged as an attractive alternative because these could reduce the use of acaricides and the costs associated with the manual labor required for animal capture, restraint, and application (10).

The development of arthropod vaccines is based on the selection of antigens that can induce an immune response in the hosts to prevent blood ingestion or the development of ticks (10). An important organ in tick physiology with many potential targets of vaccine antigens is the salivary glands (11). Ixodid ticks are hematophagous ectoparasites that spend long periods on the host (12), thus requiring the production of a cocktail of bioactive molecules in the salivary glands with important roles that guarantee the success of blood ingestion. These molecules are injected into the host skin to counteract the immune, inflammatory, and hemostatic reactions triggered at the site of the bite (13, 14). The complement system and coagulation are two examples of important mechanisms of immune and hemostatic responses, respectively, which are exerted by vertebrate hosts in blood spoliation and opposed by active molecules produced by the ticks (15).

The main function of complement inhibitors secreted in the saliva is to avoid inflammation at the bite site, prevent opsonization of salivary molecules, and protect the cells of the digestive tract (16–18). Components produced along the complement cascade, such as C3a and C5a, are strong inflammation anaphylatoxins, whereas molecules opsonized by two or three molecules of C3d can be 1,000 to 10,000 more immunogenic, respectively (19, 20). In addition, activated complement components in the intestinal environment during or after hematophagy may lead to cell lysis and, hence, tissue damage (17, 21, 22).

Hematophagous arthropods also face the mechanisms involved in hemostasis activated in the host in response to bite and blood spoliation. The fluidity of the diet is important as the tick must ingest proper amounts of blood during the feeding process to facilitate digestion. Therefore, these organisms must produce anticoagulant molecules that can oppose the blood coagulation cascade (23, 24).

Over the past few years, the molecules produced by the salivary glands of *A. sculptum* have been described in different transcriptomes (11, 25, 26); however, few of those molecules have been subjected to functional characterization. Concerning the development of tick vaccines, various studies have focused on the tick *Rhipicephalus microplus* or other tick species and there is a lack of studies concerning the selection and testing of specific antigenic targets against *A. sculptum*. Therefore, in the present study, we searched for sequences related to *A. sculptum* salivary proteins that might be involved in the feeding process and selected three proteins that underwent functional characterization and evaluation as possible vaccine antigens.

## Materials and Methods

### Experimental Ticks and Ethical Statements

*Amblyomma sculptum* ticks used in the present study were obtained from a colony maintained at the Department of Parasitology, UFMG. Specimens were kept inside incubators under semi-controlled conditions of temperature (28 ± 2°C) and humidity (90 ± 5% relative humidity). Ticks were fed on Swiss mice (*Mus musculus*) using feeding chambers according to the methodology described by Bouchard and Wikel (27). Ticks used in all experiments were 20 to 40 days after molt.

The tick colony maintenance and animal experimental procedures performed in the present study were reviewed and approved by the Ethics Committee for Animal Use at UFMG (CEUA-UFMG) under protocols 60/2020 and 103/2017.

### Target Selection

The parameters used for target selection were: putative antihemostatic activity, the presence of signal peptide, and high abundance among the protein family. For such, three transcriptomes of the salivary glands were analyzed (11, 25, 26) and the classes of salivary secreted proteins with more transcripts among the ones with putative antihemostatic function were selected. The classes of proteins with Kunitz domains and proteins of the families basic tail and 8.9 kDa were selected. Then, the most abundant transcript in each of those three classes was identified and used to search for homologous sequences in a transcriptome of the salivary glands of *A. sculptum* produced with the tick population used in the present work (not published). The transcriptome was sequenced using a methodology similar to that described by Araujo et al. (28). A pool of four salivary gland pairs were used in the transcriptome that were isolated from one unfed male and three females (one unfed, one partially fed, and one fully engorged). Three target sequences were selected and submitted to the experiments (**Supplementary Material**)

### RNA Extraction and cDNA Synthesis

RNA was extracted from pools of five larvae or two nymph whole bodies, or one female salivary gland extracted in a 0.9% saline solution using a stereomicroscope and dissecting fine tip forceps. The salivary glands or whole bodies were transferred to 1.5 ml microtubes containing 50 µl of de TRIzol® Reagent (Life Technologies®), where the total mRNA was extracted following the manufacturer’s protocol. Total RNA quality and quantity were determined using a Nanodrop™ (Thermo Scientific) and were used for cDNA synthesis using a M-MLV Reverse Transcriptase (Promega) following the manufacturer’s instructions.

### Relative Expression Analysis of Salivary Proteins

Quantitative polymerase chain reaction (qPCR) was performed in triplicate with 5 µl of Power SYBR Green PCR Master Mix Kit (Applied Biosystems), 1 µl of the cDNA previously produced as a template, 200 nM of specific primers, and Milli-Q water to achieve a final volume of 10 µl per reaction. The reactions were conducted in 96-well plates using a StepOnePlus™ System (Thermo Fisher Scientific) at 95°C for 10 min followed by 40 cycles of 95°C for 15 s and 60°C for 1 min. Relative quantification data were presented as 2^−ΔΔCt^ (29).

PCR primers for targets were designed using the Primer3 webtool (http://bioinfo.ut.ee/primer3-0.4.0/) and the sequences were as follows: AsKunitz (F - 5′ AGACGCCAACCTGCTTTCTA 3′/R - 5′ ATTGTCCCTCCTTCGCTTTT 3′), As8.9kDa (F - 5′ TCTGTACCCTCGTCGCTTTT 3′/R - 5′ AGCGTGTTACGCTCGAAGAT 3′), and AsBasicTail (F - 5′ TTTTGGGCGAAGGATAACAC 3’/R - 5′ TTTGGCTTCTTCGTGCTTTT 3′). Elongation factor 1-α (F - 5′CGTGCCCACAAAATCCTTAT 3′/R - 5′ GGAAGTCTCAAAAGCCGGTA 3′) was used as a reference gene (30).

### Cloning, Expression, and Purification of Recombinant Salivary Proteins

The target sequences were amplified by PCR and cloned into the pET28a(+) 6xHis-TEV expression vector. All primers used in the reactions were designed using the Primer3 webtool and restriction sites for *XhoI* (CTCGAG) and *NheI* (GCTAGC) were inserted in the forward and reverse ends, respectively. The final primer sequences were as follows: AsKunitz (F - 5′ CTCGAGTATAAACGGCCCAAATTTTGCT 3’/R - 5′ GCTAGCTCATGGGGCGTTGAGAATA 3′), As8.9kDa (F - 5′ CTCGAGGTCCAGGAACATGGCCACTC 3′/R - 5′ GCTAGCGTTGGTGCCATCGCAGACT 3′), and AsBasicTail (F - 5′ CTCGAGTATGATATTGTCCGTGGTTGC 3′/R - 5′ GCTAGCTTCGCCCAGGATTTTACCAT 3′). The reactions were performed using 10 µM of each primer, 1 µl of the previously synthesized cDNA, and Platinum™ Taq DNA polymerase (Invitrogen) following the manufacturer’s instructions at a 20 µl final volume mix. A Veriti™ 96-Well Thermal Cycler (Thermo Fisher Scientific) was used and the conditions were 94°C for 5 min, followed by 30 cycles of 94°C for 40 s, 60°C for 40 s, 72°C for 40 s, and a final step of 72°C for 7 s. The products were cloned into pET28a(+) 6xHis-TEV digested with *XhoI* and *NheI*. The vector was transformed following heat shock and multiplied in *Escherichia coli* DH5α cells and then purified with a Wizard® Plus SV Minipreps DNA Purification System (Promega) following the manufacturer’s specifications. The purified vectors were sequenced to confirm identity (**Supplementary Material**) and then transformed following heat shock in *E. coli* BL21. The expression was induced by adding isopropyl β-D-1-thiogalactopyranoside (0.4 mM) and shaking at 180 rpm at 37°C overnight. Purification of recombinant proteins was performed using ProBond™ Purification System (Thermo Fisher Scientific) columns with a nickel-chelating resin. Eluted fractions were analyzed with 12.5% sodium dodecyl sulfate polyacrylamide gel electrophoresis (SDS-PAGE) stained with silver nitrate solution to confirm the presence and mass weight of the recombinant proteins. Fractions containing recombinant proteins received 10 mM of 1,4-dithio-D-threitol and were dialyzed in dialysis membranes (Sigma-Aldrich®) for 48 h, with the addition of crescent volumes of phosphate buffered saline (PBS) at pH 7.4, overnight at 4°C, until a total volume of 200× the sample volume. Purified recombinant proteins were quantified (31) and stored at −20°C.

### Western Blotting of Recombinant Proteins

Western blotting was performed to confirm the expression and purification of the recombinant proteins. The proteins were separated using 12.5% SDS-PAGE and then transferred to nitrocellulose membranes at 100 V for 2 h. The membranes were blocked with 5% milk powder diluted in PBS/0.05% Tween 20 (Sigma) for 2 h and then washed three times with PBS/0.05% Tween 20. After washing, the samples were incubated with His-tag antibodies produced in mice (Sigma-Aldrich®) that had been diluted 2,000× in PBS/BSA 1% for 1.5 h. After three more washes, the membranes were incubated with peroxidase-conjugated rabbit anti-mice-IgG secondary antibodies (Sigma-Aldrich®) that had been diluted 4,000× in PBS/0.05% Tween 20 for 1.5 h. Detection was performed using a Peroxidase Substrate DAB kit (Vector Laboratories®).

### Human Plasma Recalcification Time Assay

Citrated human plasma (30 µl) was incubated with 30 µl of each recombinant protein at 0.25 µM or 0.5 µM concentration that had been diluted in PBS (pH 7.4) at 37°C for 5 min in a 96-well plate. The coagulation cascade was triggered by the addition of 30 µl of CaCl_2_ (25 mM) in each well and the absorbance was measured in a microplate reader (VersaMax™, Molecular Devices) at 650 nm with 10 s read intervals for 20 min at 37°C.

### Inhibition of Serine Protease Activity

Different concentrations of each recombinant protein were incubated with three different serine proteases in Tris buffer (20 mM Tris-HCl, 150 mM NaCl, and 0.1% BSA; pH 7.4): factor Xa (FXa) (5 × 10^−5^ U), thrombin (2.5 × 10^−3^ U), or trypsin (9 U) (Sigma-Aldrich®) at 37°C for 15 min in 0.5 ml microtubes. Then, the tube contents were placed in 96-well plates and the enzymatic reactions were triggered in the presence of the specific substrates CH_3_OCO-D-CHA-GlyArg-pNA-AcOH (Sigma-Aldrich®) for FXa, Nα-Benzoyl-L-arginine 4-nitroanilide hydrochloride (Sigma-Aldrich®) for trypsin, and N-(p-tosyl)-Gly-Pro-Arg p-nitroanilide acetate salt (Sigma-Aldrich®) for thrombin at 4 mM concentration and 100 µl final volume. Immediately after the addition of the substrate, the absorbance variation was assessed at 405 nm every 10 s with 2 s agitation between reads in a microplate reader (VersaMax™, Molecular Devices) for 30 min. The maximum reaction velocity (Vmax) values were obtained for reactions lacking inhibitors and were used as a reference for reactions in the presence of different concentrations of inhibitors.

### Complement System Hemolytic Inhibition Assays

To evaluate the effect of the salivary recombinant proteins on the classical pathway-mediated complement activation, hemolytic assays were performed using antibody-coated sheep erythrocytes (32). Erythrocytes were opsonized with IgG anti-sheep erythrocytes produced in rabbits (Sigma-Aldrich®) and diluted in GHB^+2^ solution (5 mM HEPES, 145 mM NaCl, 0.15 mM CaCl_2_, 0.5 mM MgCl_2_, and 0.1% gelatin; pH 7.4) at 2 × 10^8^ cells/ml. A total of 25 µl of normal human sera (NHS) that had been diluted 30× in GHB^2+^ and 25 µl of each recombinant protein that had been diluted in PBS at different concentrations were combined and incubated with 25 µl of diluted erythrocytes at 37°C for 30 min. To assess the hemolysis rate, 250 µl of PBS was added to each tube and the supernatants were removed after the samples were centrifuged at 1,700 × *g* for 1 min. The samples were placed in 96-well plates and evaluated using a microplate reader at 414 nm (VersaMax™, Molecular Devices). Negative controls (no serum) were subtracted from the other samples and the inhibition rates were calculated by comparing the samples containing recombinant proteins to the positive controls (samples without inhibitors). To evaluate the effect of the same proteins on the alternative pathway-mediated complement activation (33), rabbit erythrocytes diluted in Mg-EGTA (HEPES 1 mM, NaCl 30 mM, EGTA 10 mM, MgCl_2_ 7 mM, glucose 3%, and gelatin 0.02%; pH 7.4) at a concentration of 1 × 10^8^ cells/ml and NHS diluted 20× were used in the reactions.

To assess the effect of the mouse antiserum on the recombinant activities, hemolytic assays were performed as described above; however, 1 µM of each protein was incubated with its respective antiserum (produced according to the immunization procedures below) at different concentrations, at a final volume of 25 µl, before being mixed with NHS and the erythrocytes (34). Assays using rAsKunitz and rAs8.9kDa were performed with the classical pathway-mediated complement activation, whereas those with rAsBasicTail were performed using alternative pathway activation methodology. Controls were prepared by incubating each recombinant related antiserum with the erythrocytes to check whether they could cause hemolysis.

### Immunization Procedures

Swiss mice (4 to 8 weeks old) were injected subcutaneously three times at 2-week intervals with 5 µg of each recombinant protein plus 0.1 mg of aluminum hydroxide gel (Sigma-Aldrich®) as an adjuvant diluted in 100 µl of sterile PBS. Animals in the control group were inoculated only with adjuvant. Individual blood samples were collected by puncturing the tail vein of the mouse and the serum samples were obtained by blood centrifugation at 4,000 × *g* for 10 min. Samples were stored at −20°C until antibody titration assays.

### Measurement of Antigen-Specific IgG Levels in the Serum of Immunized Mice

Antigen-specific IgG levels for each salivary recombinant protein were estimated by indirect ELISA. The 96-well ELISA plates (Nunc MaxiSorp™, Thermo Fisher Scientific) were coated with 0.5 μg/well of each recombinant protein overnight. The plates were then blocked with 5% skimmed milk powder diluted in PBS/0.05% Tween 20 for 2 h at 37°C and washed with PBS/0.05% Tween 20. Serum samples diluted 1:160 in PBS/0.05% Tween 20 were added to the plates and incubated for 1.5 h. The plates were then washed and incubated with peroxidase-conjugated rabbit anti-mice-IgG secondary antibodies (Sigma-Aldrich®) that had been diluted 4,000× in PBS/0.05% Tween 20 for 1.5 h. After three washes, 100 μl of 100 mM phosphate-citrate buffer (pH 5.0) containing 0.075% H_2_O_2_ and 2 mg/ml o-phenylenediamine dihydrochloride was added to each well and the plates were incubated in a dark container for 20 min at 37°C. After incubation, 100 μl of 2 M H_2_SO_4_ was added to stop the reaction and the IgG levels were estimated with a 492 nm read on a microplate reader (VersaMax™, Molecular Devices).

### Tick Infestation and Vaccine Efficacy Calculations

Two weeks after the third injection of the immunization protocol, all mice were prepared with a feeding chamber attached to the dorsal region (27) and infested with one adult tick couple or 10 nymphs per mouse. The levels of protection were determined by measuring the following feeding parameters: mortality that occurred during blood feeding and before oviposition or molt, the engorged tick weight, and the feeding period for nymphs and females. To assess the reproductive parameters, measurements were taken of the egg mass weight and percentage of eggs hatching. Vaccine efficacy rates against female parasitism were calculated according to the following formula: (%E) = 100 [1−(CRT × CR0 × CRF)], which comprises the coefficient of reduction in the number of engorged female ticks in the vaccinated/control group (CRT), coefficient of reduction in oviposition in the vaccinated/control group (CR0), and coefficient of reduction in egg fertility in the vaccinated/control group (CRF) (35).

## Results

### Selection and Expression Levels of the Tick Salivary Transcripts

A transcript coding for a protein of 8.7 kDa containing a Kunitz_bovine pancreatic trypsin inhibitor domain and that was 99% identical to the “putative tick Kunitz 78” (accession: JAC23688.1) was selected. This protein was named AsKunitz.

A transcript coding for a 9.8 kDa protein with a Von Willebrand factor (vWF) type c domain was also selected. The protein has eight conserved cysteine characteristics of the 8.9 kDa tick superfamily. Its mature sequence was identical to the “hypothetical protein (*Amblyomma cajennense*)” (accession: JAC23736.1). This protein was named As8.9kDa.

A 16.7 kDa protein was also selected. It has several lysine residues in the carboxy terminal region, three disulfide bonds, and a YY block, which are characteristics of the Basic Tail protein family and are exclusive to ticks. The protein was 86% identical to a sequence named “putative basic tail protein (*A. cajennense*)” (accession: JAC23973.1). This protein was named AsBasicTail.

**Figure 1:**
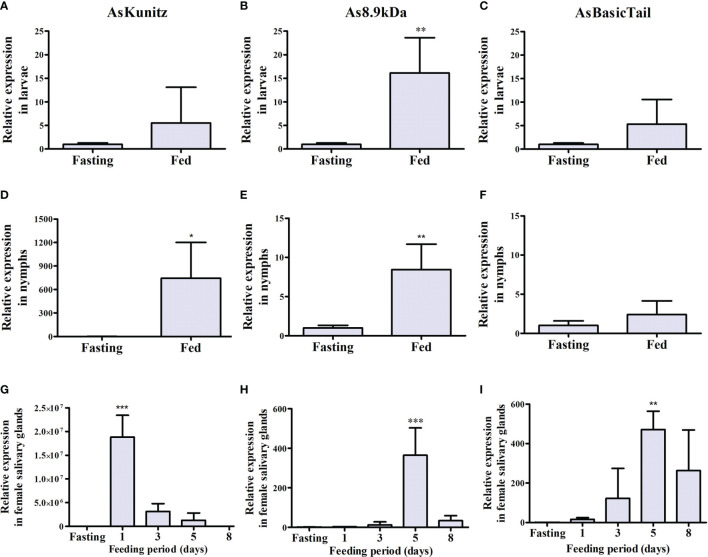
Relative messenger RNA (mRNA) levels of AsKunitz, As8.9kDa, and AsBasicTail in *Amblyomma sculptum* instars during feeding. Measurements were performed using quantitative polymerase chain reaction in whole body for larvae **(A–C)** and nymphs **(D–F)** and in the salivary glands for females **(G–I)**. Feeding = 3 days of feeding. Statistical analysis: **(A–F)**—t-test, **(G–I)**—ANOVA–Dunnett, *p < 0.05, **p < 0.01, and ***p < 0.001 indicate difference from the fasting group. Data is shown as mean ± standard deviation (SD).

Expression of the three selected transcripts was observed in the larvae, nymphs, and adults of *A. sculptum* at different levels during feeding. In general, transcripts were expressed at basal levels in the unfed ticks and their expression increased as feeding started (**Figure 1**). For immature tick instars, the highest expression was observed in larvae after feeding, with As8.9kDa expression significantly (p < 0.05) increasing by ~15-fold (**Figure 1B**). For nymph feeding, the expression of AsKunitz significantly increased (p < 0.05) by ~750-fold (**Figure 1D**).

In adult females, the profile of the mRNA expression in the salivary glands during feeding was similar between As8.9kDa and AsBasicTail, which had peaks of expression close to the end of the feeding (at day 5 of feeding) (**Figures 1H, I**). AsKunitz was upregulated at the beginning of blood feeding (**Figure 1G**). When the expression level was analyzed at the peak times, AsKunitz was the most abundant with mRNA levels that were almost 20 million times higher than those in the unfed ticks (**Figure 1G**). As8.9kDa and AsBasicTail also had a significant (p < 0.05) upregulation; however, their mRNA levels were 365- and 471-fold higher than those in the fasting ticks (**Figures 1H, I**).

### Recombinant Expression and Functional Characterization of Selected Salivary Proteins

**Figure 2:**
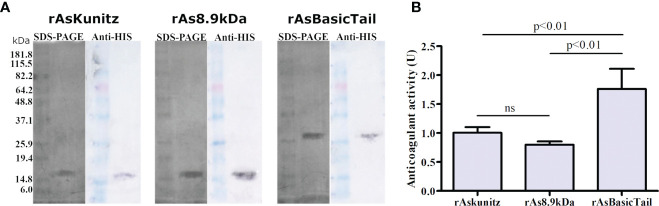
Expression of rAsKunitz, rAs8.9kDa, and rAsBasicTail and their effect on the coagulation cascade. Sodium dodecyl sulfate polyacrylamide gel electrophoresis (SDS-PAGE) (silver staining) and western blotting (developed using anti-His-tag antibodies) of each recombinant protein **(A)**. Effect of 0.25 µM of each recombinant on the human coagulation cascade **(B)**. One unit (U) of anticoagulant activity indicates that the coagulation time was doubled. Statistical analysis: ANOVA–Tukey, ns = not significant (p>0.05). Data is shown as mean ± SD.

To assess the biological activities of these three proteins, they were expressed as recombinant proteins in *E. coli*. Recombinant proteins appeared on SDS-PAGE and western blotting with molecular weights approximately twice their estimated size (**Figure 2A**).

The three recombinant proteins prolonged the coagulation time, with higher activity for rAsBasicTail (**Figure 2B**). These results confirm that the recombinants were correctly folded and active. Coagulation was completely inhibited (no clots formed during the assay period) when 0.5 µM of each recombinant was used.

**Figure 3:**
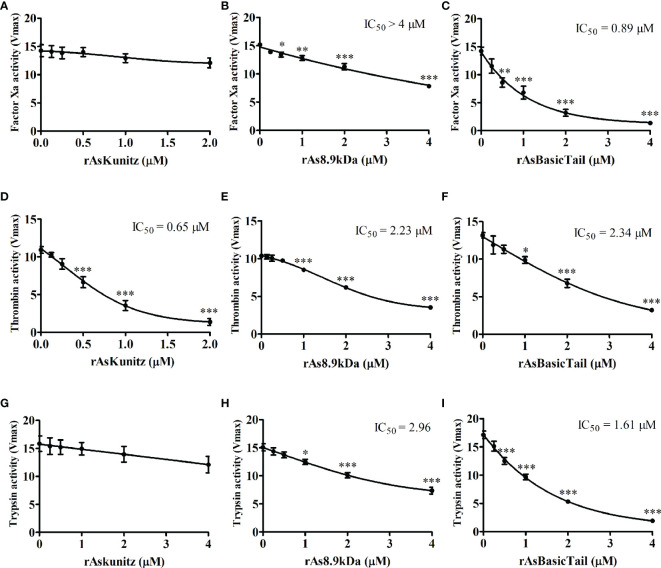
Effect of the recombinant proteins on the activity of factor Xa **(A–C)**, thrombin **(D–F),** and trypsin **(G–I)**. Data is shown as mean ± standard error (SE). Statistical analysis: ANOVA–Dunnett, *p < 0.05, **p < 0.01, and ***p < 0.001 indicate difference from the sample without recombinants (0 µM).

The coagulation cascade has several serine proteases; therefore, we checked the ability of the recombinant proteins to act on FXa, thrombin, and trypsin. rAsKunitz was specific for thrombin, where it had a significant inhibitory activity (IC_50_ = 0.65 µM) and had no effect on FXa and trypsin (**Figures 3A, D, G**). rAsBasicTail and rAs8.9kDa significantly affected (p < 0.05) the activity of all serine proteases tested, but with distinct levels of activity. Inhibition promoted by rAsBasicTail was stronger on FXa and trypsin (**Figures 3C, F, I**), whereas rAs8.9kDa showed higher inhibition of thrombin (**Figures 3B, E, H**).

**Figure 4:**
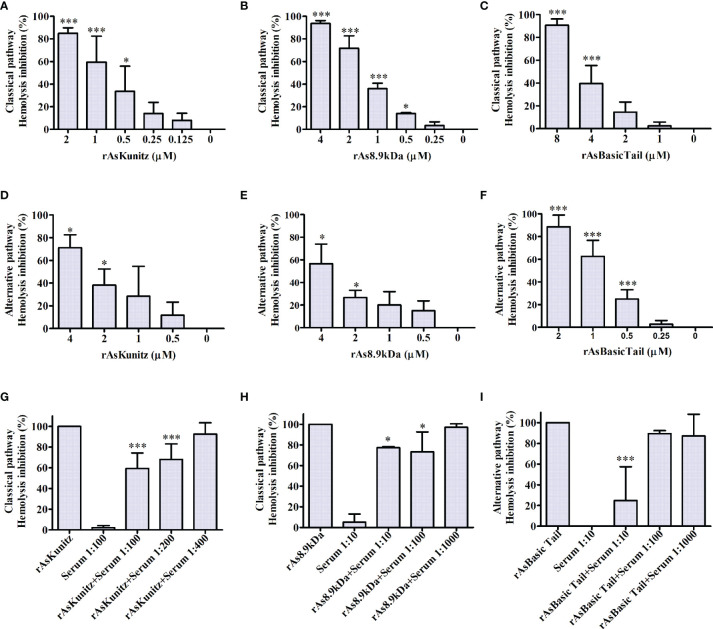
Effect of rAsKunitz, rAs8.9kDa, and rAsBasicTail on the classical **(A–C)** and alternative **(D–F)** pathways of the human complement system. Recombinant proteins were incubated with the respective mice antiserum before being added to the classical **(G, H)** or the alternative **(I)** pathway assays. Data is shown as mean ± SD. Statistical analysis: ANOVA–Dunnett, *p < 0.05 and ***p < 0.001. In **(A–F)**, asterisks indicate statistical difference from controls, i.e., 0 µM in **(A–F)** and the sample without specific antiserum in **(G–I)**.

Recombinant proteins also affected the human complement cascade. All three proteins inhibited the activation of the complement system in both the classical and alternative pathways. rAsKunitz and rAs8.9kDa were more efficient in inhibiting the classical pathway (**Figures 4A–C**), whereas rAsBasicTail was the best inhibitor of the alternative pathway (**Figures 4D–F**), achieving more than 80% hemolysis inhibition with 4 µM. To confirm the complement inhibition activity, the assays were repeated, but the recombinants were incubated with mice specific anti-sera before being added to the other components of the assay. Each recombinant was tested in a pathway that promoted more inhibition and the results showed that the antibodies could partially and significantly (p < 0.05) impair their inhibition of the complement system by all three recombinants (**Figures 4G–I**).

### Effect of Recombinant Proteins as Vaccine Antigens Against Tick Infestation

**Figure 5:**
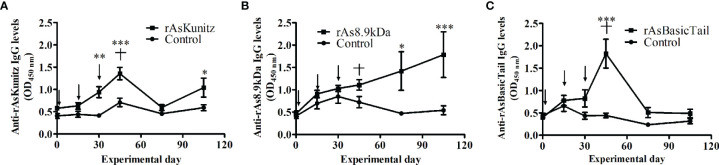
Profile of specific serum IgG levels in mice vaccinated with rAsKunitz **(A)**, rAs8.9kDa **(B),** and rAsBasicTail **(C)**. Serum IgG levels against each of the recombinant were accessed by ELISA. Arrows indicate the three injections used for immunization and the cross indicates the challenge with ticks. Statistical analysis between control and vaccinated groups: Two-way ANOVA–Bonferroni, *p < 0.05; **P < 0.01; ***p < 0.001.

To assess the potential of the proteins as vaccine antigens, the mice were immunized with each recombinant and used as a feeding source for nymphs and females of *A. sculptum*. Immunization induced two distinct profiles of specific IgG levels (**Figure 5**). Mice immunized with rAsKunitz and rAsBasicTail had an increase in specific IgG levels until the challenge followed by a gradual decrease (**Figures 5A, C**), whereas the specific anti-rAs8.9kDa IgG levels increased gradually until the end of the experiment (**Figure 5B**).

**Figure 6:**
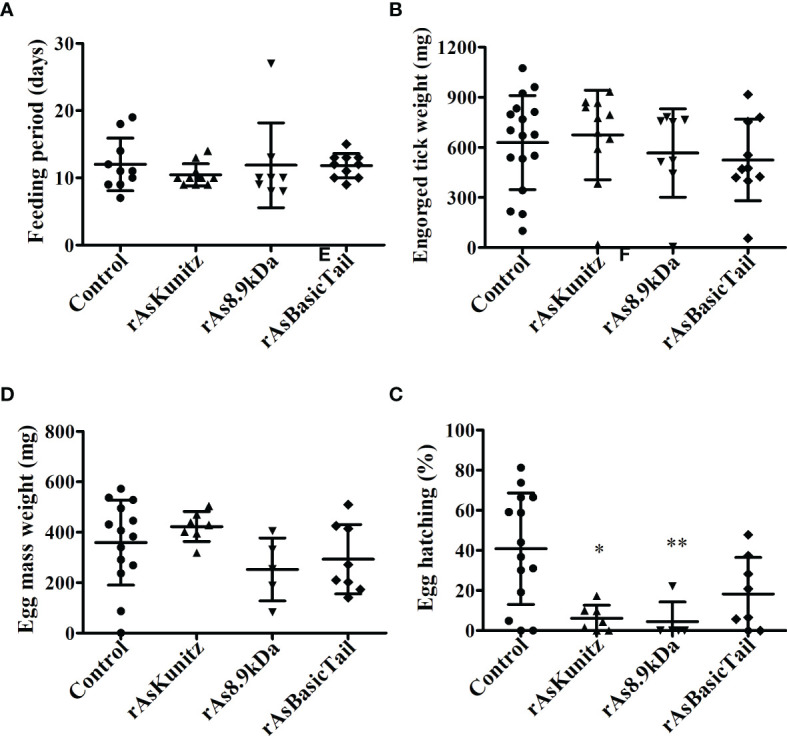
Feeding **(A, B)** and reproductive **(C, D)** parameters of *Amblyomma sculptum* females fed on mice previously immunized with rAsKunitz, rAs8.9kDa, or rAsBasicTail. Data is shown as mean ± SD. Statistical analysis: Kruskal-Wallis–Dunn’s, *p < 0.05 and **p < 0.01 indicate significant difference from controls.

When females were fed on mice previously immunized with each recombinant protein, no significant (p > 0.05) effects were observed on the feeding period, engorged tick weight, and egg mass laid by females (**Figures 6A–C**). However, mortality and egg hatching were considerably affected in females from the groups fed on immunized mice. Mortality reached up to 58% when the nymphs were fed on mice that had been previously immunized with the recombinant proteins compared to a rate of 30% for the control group (**Table 1**) and egg viability was at least 50% lower than that of the control groups (**Figure 6D**). rAs8.9kDa induced higher female mortality by 94% (**Table 1**) and rAsKunitz and rAs8.9kDa both induced more than 80% fewer egg hatching than that of the control group (**Figure 6D**). The efficacy of the recombinant proteins as antigens showed that immunization with rAsKunitz and rAs8.9kDa had the highest effects (reduced infestation by at least 85%), whereas immunization with rAsBasicTail was only 59.4% lower than that of the control group (**Table 2**).

**Table 1:** Mortality of *Amblyomma sculptum* females and nymphs fed on mice previoulsly immunized with rAsKunitz, rAs8.9kDa, and rAsBasicTail.

Instar/Group	Control	rAsKunitz	rAs8.9kDa	rAsBasicTail
Female mortality*	30.0	41.6	58.3	33.3
Nymph mortality*	32.0	70.0	100.0^+^	100.0^+^

*Data show the percentage of ticks that died among the total specimens used in each group.

^+^All nymphs from the rAs8.9kDa and rAsBasicTail groups died while attached to the host feeding.

**Table 2:** Efficacy of vaccination of mice with rAsKunitz, rAs8.9kDa, or rAsBasicTail against *Amblyomma sculptum* ticks.

** **	****rAsKunitz	rAs8.9kDa****	rAsBasicTail****
**CRT^a^**	1.07	0.78	0.98
**CRO^b^**	0.90	0.53	0.79
**CRF^c^**	0.15	0.17	0.52
**Efficacy (%)^d^**	85.3	92.8	59.4

^a^Coefficient of reduction in the number of engorged female ticks in the vaccinated/control group (CRT).

^b^Coefficient of reduction in oviposition in the vaccinated/control group (CR0).

^c^Coefficient of reduction in egg fertility in the vaccinated/control group (CRF).

^d^Vaccine protection against ticks considering the effects on CRT, CTO, and/or CRF. Percentage of efficacy (%E) = 100 [1−(CRT × CR0 × CRF)].

**Figure 7:**
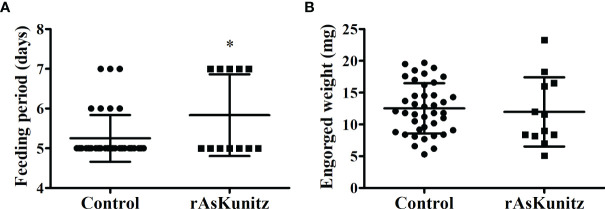
Feeding parameters of *Amblyomma sculptum* nymphs fed on mice previously immunized with rAsKunitz. Duration of blood feeding **(A)** and tick weight after detachment **(B)**. Data is shown as mean ± SD. Statistical analysis: Mann-Whitney, *p < 0.05.

The vaccination experiments performed with the nymphs also had high efficacies, with mortality being the most affected parameter. The control group had 32% mortality, whereas all the nymphs died after feeding on mice immunized with rAs8.9kDa and rAsBasicTail, and 70% died when fed on rAsKunitz-immunized mice (**Table 1**). All the nymphs from the rAs8.9kDa and rAsBasicTail groups died while attached to the host feeding. Nymphs of the rAsKunitz group that survived required significantly (p < 0.05) more days to complete feeding (**Figure 7A**); however, they had final weights similar to those of the control group (**Figure 7B**).

## Discussion

The present study characterized three salivary molecules from *A. sculptum* and evaluated their potential as vaccine antigens for the development of novel tick control methods. All three proteins showed antihemostatic and anti-complement activities and promising anti-tick efficacies, which make them potential vaccine antigens against *A. sculptum*. The results also provide information on the biological activities of proteins belonging to three families of proteins. Despite being relatively common in tick salivary glands (13, 36), functional studies on members of the 8.9 kDa and Basic Tail families are scarce.

AsKunitz, As8.9kDa, and AsBasicTail were expressed in all tick instars and presented upregulation, with distinct levels, after feeding started. The larvae and nymphs had lower levels of upregulation that varied from 2.4- to 745-fold, whereas the variation in adults, at peak production, was considerably higher and ranged from 365- to almost 20 million-fold. The larvae and nymphs were analyzed at day 3 of feeding and, therefore, the peak production of each protein might not have been assessed.

In the female salivary glands, the expression profile showed that AsKunitz was more highly expressed at the beginning of feeding, whereas As8.9kDa and AsBasicTail were more important at the end of feeding. Several studies have shown that the levels of salivary secreted molecules may vary with tick feeding (11, 37–40); however, further studies are required to elucidate the meaning of such profiles. In the study by Esteves et al. (11), the expression of secreted proteins in the salivary glands of *A. sculptum* increased from 11 to 36% at 72 h after the beginning of hematophagy and several genes from the Kunitz, 8.9kDa, and basic tail families were among the most upregulated. These results are in agreeance with the results from our study and indicate that these proteins are important for blood feeding with an expression profile that might be useful in a multicomponent vaccine because tick salivary molecules will be attacked by the immune system of vaccinated hosts during most of the hematophagy period.

The recombinant expression produced proteins with higher molecular weights (MW) on SDS-PAGE than expected. Once their identity was checked by sequencing and anti-His western blotting, it was possible that the His-tag influenced the protein mobility in the SDS-PAGE or that recombinant proteins formed multimers, as discussed in previous studies (33, 41, 42).

The biological assays undertaken with the recombinant proteins revealed that they were all efficient in delaying the coagulation time of the blood plasma, suggesting that they are inhibitors of serine proteases. They could act on serine proteases, but with different activities and specificities. rAsKunitz was more specific with inhibition only on thrombin activity, whereas rAs8.9kDa and rAsBasicTail showed more general activity, acting on the three serine proteases tested.

Thrombin inhibitors with Kunitz-like domains have been previously identified and characterized in other species of ixodid ticks, such as the intestinal inhibitors boophilin, hemalin, and ixophilin, which have been described in *R. microplus*, *Haemaphysalis longicornis*, and *Ixodes scapularis*, respectively (43–45). The protein amblin also inhibits thrombin; however, it is present in the hemolymph of *Amblyomma hebraeum* (46). AsKunitz is the first Kunitz protein to show thrombin inhibitory activity from a tick salivary gland. Unlike the previously mentioned inhibitors, which have two Kunitz domains, AsKunitz has only one and has a considerably smaller MW than the others. Nevertheless, rAsKunitz showed efficiency in doubling the time needed for plasma coagulation similar to that of recombinant hemalin (43) and compatible with that observed for the native boophilin (44).

The AsKunitz mechanism of action remains unknown; however, it appears to be related to that of amblin (46) because both have a basic nature (amblin has a pI of 9.7) and the presence of two cysteine residues in the carboxy-terminal region, which may be responsible for the formation of another disulfide bond. This, in turn, could interfere with the exosite I binding region of thrombin (47).

rAsKunitz as well as the other Kunitz proteins cited above could not inhibit FXa. However, other salivary FXa inhibitors of the Kunitz family have already been described as amblyomin-X from *A. sculptum* (48) and Ixolaris from *I. scapularis* (49). Ixolaris has two Kunitz domains and is a non-competitive inhibitor of FXa exosite. The tick anticoagulant protein (TAP), a salivary inhibitor of the argasid tick *Ornithodoros moubata* (50), has only one Kunitz domain similar to rAsKunitz and can inhibit FXa. However, the TAP mechanism of action is linked to the presence of a tripeptide (Tyr–Asn–Arg) in the amino terminal region, which is absent from rAsKunitz and acts by blocking the active site of the enzyme (47).

rAs8.9kDa was the recombinant with the lowest inhibition of the coagulation cascade. Although it could significantly inhibit the enzymatic activity of FXa, thrombin, and trypsin, but the IC_50_ achieved indicates that inhibition occurred with low efficiency. These findings indicate that As8.9kDa is not a specific inhibitor for any of the serine proteases tested and that it may play a role in *A. sculptum* hematophagy other than the inhibition of the coagulation cascade. Coagulation inhibitors have not yet been described in the 8.9 kDa family of proteins.

rAsBasicTail was the recombinant with the greatest effectiveness in inhibiting the catalytic activity of FXa. This was expected because the basic tail family has members previously characterized as FXa inhibitors, such as the 11.8 kDa salivary protein Salp14 from *I. scapularis* (51). *I. scapularis* Ixonnexin is also a basic tail protein that interacts with FXa (52). AsBasicTail has characteristics similar to those of Ixonnexin, such as a tail rich in lysine residues and the presence of three disulfide bonds responsible for maintaining its secondary structure. Although the AsBasicTail mechanism of action was not investigated, the interaction with FXa might be related to the presence of the basic carboxy-terminal region because proteins with structures similar to those of the tick salivary lectin pathway inhibitor and the recombinant Salp9, which do not have this structure, did not show anticoagulant activity (51, 53).

The hemolytic assays demonstrated that the salivary recombinants, tested separately, inhibited the activation of the human complement system in both the classical and alternative pathways. Although anti-complement activity has already been observed in the saliva of *A. sculptum* (32), anti-complement molecules have not been identified until the present study.

rAsKunitz and rAs8.9kDa blocked the activation of the classical pathway and are the first complement inhibitors described for ticks in their respective families. The complement factor inhibited by rAsKunitz still requires further studies to be elucidated; however, the different inhibition promoted in the classical and alternative pathways might suggest that more than one component of the cascade was affected. The anti-complement activity observed in rAs8.9kDa, in turn, may be related to the presence of the vWF domain in its sequence, as vWF acts as a cofactor in the regulation of the complement system in vertebrates via the cleavage of C3b (54). Thus, rAs8.9kDa could also be acting in the inactivation of C3b and, consequently, inhibiting the activation of the cascade.

AsBasicTail strongly opposed the activation of the complement by the alternative pathway. It is the first inhibitor of *A. sculptum* and the first molecule of the Basic Tail family, which is described as an inhibitor of the alternative pathway. However, it is not the first complement inhibitor characterized in the family because the tick salivary lectin pathway inhibitor is also a Basic Tail protein and can block the activation of the lectin pathway (53).

In addition to the hemolytic assays, the results showed that specific antibodies present in the serum of animals immunized with each of the recombinant salivary proteins could interfere with their anti-complement activity, probably due to opsonization by the antibodies. Similar results were observed in hemolytic assays with SALO and lufaxin, the salivary complement inhibitors of *Lutzomyia longipalpis* (33, 34). The importance of the complement in host resistance against ticks has been previously shown (21, 55, 56) and this effect is highly desirable if the proteins are intended to be used as vaccine antigens. Salivary molecules of hematophagous arthropods also act in the intestine (57); therefore, the suppression of anti-complement activity could contribute to the activation of the complement cascade in the intestinal lumen, culminating in lesions on the epithelial cells (17) and, consequently, loss of digestive and reproductive efficiency or even death of the parasite (58). Additionally, it has been observed that blocking the activity of salivary proteins with anti-complement function can impair the transmission of pathogens to vertebrate hosts (53, 59–61); thus, the immunization of hosts with the salivary antigens of *A. sculptum* could also interfere with the transmission of *R. rickettsii*.

The simultaneous presence of anticoagulant and anti-complement activities performed by *A. sculptum* recombinant proteins is not uncommon in serine protease inhibitors produced by other invertebrates. Salivary proteins from *L. longipalpis* and *Amblyomma americanum* were previously shown to be able to delay plasma clotting time and block the activation of the complement system (34, 62, 63). This may be related to the non-specific inhibition of different serine protease members of the cascades. In natural circumstances, the effect may be even higher once it is known that both systems are intrinsically related. Components of the coagulation cascade, such as FXa and thrombin, can act in the cleavage of complement factors C3 and C5, contributing to their activation (64). Thus, we can infer that the salivary proteins addressed in the present study may be related to various functions in the parasite-host interaction, which encourages further studies of functional characterization.

Vaccine trials using recombinant proteins as antigens showed promising efficacy against *A. sculptum*, especially rAs8.9kDa and rAsKunitz. The tick mortality rate was the first parameter affected by the vaccine. It increased in groups of both females and nymphs fed on vaccinated mice; however, it was much greater for nymphs and reached 100% for two recombinant proteins. It is not clear why the mortality rate was higher in nymphs; however, the cause may be explained by the lower expression levels of the native proteins. Lower levels of the inhibitors could lead to a more expressive blocking of their activity by the antiserum of vaccinated mice.

The vaccinations did not decrease the oviposition of engorged females, which was similar to that found by Andreotti et al. (65). However, the great variation in the hatching rate of the eggs was the main parameter affected by the vaccine. One possible explanation would be the influence of feeding on immunized hosts on the tick microbiota. In this case, the ingested blood containing antibodies against anti-complement molecules could increase the complement system activation in the intestinal environment, thereby reducing the symbiotic microbiota of the arthropods, culminating in the reduction of reproductive fitness. Previous studies have shown the importance of the intestinal microbiota in tick fertility. Zhong et al. (66) demonstrated that the oviposition and fertility of eggs of engorged females of *A. americanum* treated with antibiotics were significantly decreased. Machado-Ferreira et al. (67) evaluated the bacteria present in the eggs of *A. sculptum* and showed that the microbiota can act as a chemical defense and would be beneficial for its development in the environment.

Vaccine trials against ticks of the *A. cajennense* complex are rare in the literature. The BM86 antigen, which is the base of the commercial vaccines against *R. microplus*, was ineffective against nymphs and adults of *A. cajennense* sensu lato when calves were used as hosts (68). In an experiment conducted with larvae on rabbits, the tick P0 peptide showed 54% efficacy against *Amblyomma mixtum*, which is another species of the *A. cajennense* complex (69).

Several research groups are looking for an efficient anti-tick vaccine. Studies have shown that results vary; however, some candidates have reached up to 97% efficacy (70, 71). The efficacies obtained here for rAs8.9kDa and rAsKunitz were superior to those described in studies with *A. cajennense* s.l. and other trials that tested antigens against other species of ticks (70, 71). However, mice were used in the present study, which are not natural hosts of *A. sculptum* and the results might be divergent if natural hosts were used in the vaccine trials. In studies that tested salivary antigens against ticks in their natural hosts, such as *R. microplus* on cattle, considerably lower efficacies were found, as seen for the 32% efficacy from a thrombin inhibitor of the Kunitz family (65), 73.2% for a multicomponent vaccine containing a serine protease inhibitor (72) and 60% for a salivary metalloprotease (73).

The activities of the proteins shown in the present study are also in agreeance with the host concern. If coagulation and complement inhibition are truly related to vaccine efficacy, it is important to check their activity when using plasma/sera from natural hosts (human material were used here) as activity may vary significantly when samples from different animal species are used (74, 75).

The data obtained here are encouraging for further studies to be performed with AsKunitz, As8.9kDa, and AsBasicTail, mainly for the evaluation of the efficacy against *A. sculptum* on other hosts and to verify a possible interference on the transmission of *R. rickettsii* by the vectors (76). In addition, tests with formulations containing antigenic combinations should be performed to increase the efficacy of the vaccine, which would be another step in the search for a commercial vaccine against *A. sculptum*.

## Data Availability Statement

The raw data supporting the conclusions of this article will be made available by the authors, without undue reservation.

## Ethics Statement

The animal study was reviewed and approved by Comissão de Ética no Uso de Animais (CEUA) from the Federal University of Minas Gerais (UFMG).

## Author Contributions

GC and IR—performed the experiments, and analyzed and discussed the data. NG, MS, MP, GP, LK, and RA were involved in procedures related to colony maintenance, evaluation of relative mRNA levels, and production of recombinant proteins. FO, JV, DB, NG, and RA conceived and designed the procedures to select protein targets and characterize the activity of the recombinants. OJ, RG, RF, and RA conceived and designed the vaccination trials. GC, IR, and RA wrote the paper. RNA supervised and coordinated the experiments. All authors contributed to the article and approved the submitted version.

## Funding

This research was supported in part by the Intramural Research Program of the National Institutes of Health (NIH), National Institute of Allergy and Infectious Diseases (FO, JGV), and the Brazilian Agencies Fundação de Amparo à Pesquisa do Estado de Minas Gerais (FAPEMIG), Conselho Nacional de Desenvolvimento Científico e Tecnológico (CNPq), Coordenação de Aperfeiçoamento de Pessoal de Nível Superior (CAPES), Pró-Reitoria de Pesquisa (PRPq/UFMG),and Instituto Nacional de Ciência e Tecnologia em Entomologia Molecular (INCT-EM).

## Conflict of Interest

The authors declare that the research was conducted in the absence of any commercial or financial relationships that could be construed as a potential conflict of interest.
