# Supplementary material for: Amblyomma sculptum Salivary Protease Inhibitors as Potential Anti-Tick Vaccines
Source: Front Immunol. 2021 Feb 4;11:611104. doi: 10.3389/fimmu.2020.611104 (PMC7901972; doi:10.3389/fimmu.2020.611104)
Supplement: Supplementary file 1 [file DataSheet_1.docx]

# SUPPLEMENTARY MATERIAL

**A**

**AsKunitz**

**MKTPTCFLCALLCLMVVLVQG**YKRPKFCYAKAKEGQCGHERPSIERWYFDARYGYCGPFLWGGCGGNNNNFPNCTLCMTVCSDHSDPEGACRNILNAP

**As8.9kDa**

**MRAFTASFCTLVAFATVIC**DVQEHGHSYLTRNVTVENGACIFERNTLPDGETKALHDPCVIATCYAARREVNATLCRNFGVDPGCRVQWTPDGVYPQCCPRQVCDGTN

**AsBasicTail**

**MVLLLFLSIFLLNEVHG**DYDIVRGCPPKDAQGSVVEDCNYYCNHTHEGYYVNGTRCKITHIPGVTEGVCIDLLGMEGCHPPNDTFARRFMKFWVKDNTTPTTGTQETKPTTVATTTTTQSSTTTKSKSTKKPKKTKKTKRPKTKRPKTSKKNKTKTSTKPTTEFSW

**B**

**rAsKunitz**

**MGHHHHHHENLYFQGHMAS**YKRPKFCYAKAKEGQCGHERPSIERWYFDARYGYCGPFLWGGCGGNNNNFPNCTLCMTVCSDHSDPEGACRNILNAP

**rAs8.9kDa**

**MGHHHHHHENLYFQGHMAS**VQEHGHSYLTRNVTVENGACIFERNTLPDGETKALHDPCVIATCYAARREVNATLCRNFGVDPGCRVQWTPDGVYPQCCPRQVCDGTN**LEHHHHHH**

**rAsBasicTail**

**MGHHHHHHENLYFQGHMAS**YDIVRGCPPKDAQGSVVEDCNYYCNHTHEGYYVNGTRCKITHIPGVTEGVCIDLLGMEGCHPPNDTFARRFMKFWVKDNTTPTTGTQETKPTTVATTTTTQSSTTTKSKSTKKPKKTKKTKRPKTKRPKTSKKNKTKTSTKPTTEFSW

**Supplementary figure 1:** Amino acid sequences of *Amblyomma sculptum* salivary proteins. **(A)** Sequences of native proteins. Signal peptides, as detected by the signalP-5.0 tool, are shown in bold. **(B)** Sequences of recombinant proteins. Amino acids codified by vector codons are shown in bold.
